# Professional and personal experiences of workplace violence among Italian mental health nurses: A qualitative study

**DOI:** 10.3934/publichealth.2024059

**Published:** 2024-12-06

**Authors:** Ilenia Piras, Igor Portoghese, Massimo Tusconi, Federica Minafra, Mariangela Lecca, Giampaolo Piras, Paolo Contu, Maura Galletta

**Affiliations:** 1 Clinical Trials Sector, General Affairs, ‘A. Cao’ Microcythemic Hospital, ASL Cagliari, Cagliari, Italy; 2 Department of Medical Sciences and Public Health, University of Cagliari, Monserrato, Italy; 3 Department of Psychiatry, University of Cagliari, Cagliari, Italy; 4 Nursing Home Gersia, Selargius, Italy; 5 Surgical Department, SS. Trinità Hospital, ASL Cagliari, Cagliari, Italy; 6 Sardinia Regional Health Emergency Service, Cagliari, Italy

**Keywords:** workplace violence, nurses, mental health care, qualitative study, health consequences for workers

## Abstract

**Background:**

Violence against healthcare workers in psychiatric settings is a concern in the literature. Violence effects for healthcare professionals and organizations include an absence from work due to injury or illness, a decreased job satisfaction, and a lower quality of work. The aim of this study is to identify the consequences of violence on the health, work habits, and performance of nurses working with psychiatric patients.

**Methods:**

The study was conducted using semi-structured interviews with 18 nurses from different hospitals and territorial psychiatric settings in Southern Italy. The interviews were conducted from July to December 2020 by telephone and were recorded with the consent of the participants. The collected data were transcribed and analyzed.

**Results:**

The narratives revealed five main themes: (1) Feelings about the violence experienced; (2) the effects of violent incidents on nurses; (3) features of the mental health setting related to the phenomenon of the assault; (4) the care and organizational aspects to prevent the assault; and (5) the care in psychiatric settings during the COVID-19 pandemic. The aggressions resulted in a change in the nurses' work habits and performances; they were more careful after the aggression and modified their approach to the patient. Additionally, the nurses discussed developing skills and strategies to protect themselves and avoid aggression.

**Conclusions:**

Aggression has a negative impact on the health and work performance of nurses. Adopting personal and nursing strategies in place to prevent aggression allows them to improve the patient care and to protect themselves from such incidents. The creation of a safer work environment by healthcare organizations in which professionals work can improve their health, job performance, and the effectiveness of psychiatric nursing care.

## 1. Introduction

Violence in the workplace has major consequences on the lives of workers, the productivity of healthcare organizations, and the quality of care. Although the global community has made it clear that violence and harassment in the workplace is not tolerated and must end [1], violence continues to occur in hospital and community settings. In particular, in hospitals, it occurs more frequently in psychiatric wards, emergency rooms, waiting rooms, and geriatric wards [2]. Although numerous recommendations of world organizations support the need to achieve the elimination of violence in the workplace [3]–[5], it is difficult to find data that represents the actual situation. In fact, complaints are often only made when an assault on the staff generates an injury. In the case of verbal aggression, the worker often renounces the complaint. Among health care workers, nurses are at the highest risk of violence in the workplace [6]. The consequences on their health and work can be very significant. The aggressor may be the patient, the carer, another user, or another worker. The most common causes of patient violence are mental illness, acute intoxication, and substance use [7]. No less important are the predisposing factors for episodes of violence related to healthcare facilities, such as overcrowding, the number of professionals present, the quality and quantity of the staff trainings, and the use of restrictive measures [8]. In psychiatric wards, inpatient aggression often occurs without a clear motivation and in an unpredictable and unexpected manner. In addition to the patient's unstable mental state, unmet needs and social conflicts may also be motivating factors [9].

### 1.1. Violence during COVID-19 pandemic

The COVID-19 pandemic has exacerbated workplace violence incidents in the healthcare sector. Some meta-analyses [10],[11] have reported that violence in healthcare settings increased from the middle of the first pandemic period to the end of the pandemic, with a greater increase in incidents of verbal violence compared to physical violence. Contributing factors included family visit restrictions, long waiting times for treatment, and a lack of resources and staff [12]. To the best of our knowledge, little is known about how violence has changed in mental health settings during the COVID-19 pandemic. In Italy, legislation was also enacted in the year 2020 to ensure the safety of healthcare and socio-healthcare professionals in the performance of their duties; to this end, the Ministry of Health set up the National Observatory on the safety of healthcare and socio-healthcare professionals [13],[14]. Previously, the Ministry of Health issued a recommendation to prevent acts of violence against healthcare workers and to encourage the analysis of workplaces, related risks, and the adoption of initiatives and programs to prevent acts of violence and/or mitigate their negative consequences in all hospital and territorial healthcare facilities [15]. Priority was given to activities considered at a higher risk, including hospital and community psychiatric services. Although several previous studies have investigated violence in the workplace and its consequences, it is necessary to deepen nurses' experiences regarding this important issue to improve the knowledge in this area, particularly in the Italian context.

### 1.2. Aim of the study

The aim of this study is to explore the experiences of violence among nurses caring for patients with mental health problems and to identify the consequences for their work habits and nursing practices. The possible influence of the COVID-19 pandemic on the violence experienced by nurses in the workplace and the reasons why its frequency may have changed in psychiatric care settings are also investigated.

## 2. Materials and methods

### 2.1. Study design and procedures

The qualitative study involved semi-structured interviews with nurses that cared for individuals with mental health difficulties in hospital and community settings. The research was conducted in Italy from July to December 2020. The potential participants were identified through telephone interviews with services that cared for people with mental health problems in hospital and community settings; the participants anonymously offered to take part in the study. Recruitment was carried out by telephone by a member of the research team, who first explained the aim of the study and the modalities of participation to any nurse who showed an interest in sharing their patient care experience in a psychiatric setting. Nurses agreed to the terms of participation and provided informed consent. They provided their telephone numbers and then were contacted for the interview. The inclusion criteria were as follows: (1) be a nurse who cared for people with mental health problems in hospital and community settings at the time of the interview; (2) have worked as a nurse in a mental health setting for at least 1 year; and (3) freely consent to participate in the study.

#### 2.1.1. Tool

Based on the purpose of the study, a written semi-structured interview guide (Table 1) was created from the available literature [16]–[25]. This guide aimed to elicit the nurses' experiences of violence and to identify the consequences on their work habits and nursing practice. During the interviews, follow-up questions were also used to better understand the interviewee's narrative. In general, the interview questions were addressed to the nurses in a flexible and functional manner as the interview flowed.

**Table 1.** Study interview guide.

**Study interview guide**
1) What does workplace violence mean to you?
2) How do you feel about violence at work?
3) What are the consequences of violence in the workplace?
4) Was there any violent or aggressive behavior during the COVID-19 pandemic?
5) What do you think can be done to prevent violence?

#### 2.1.2. Data collection

Twenty-four potential participants met the inclusion criteria. The nurses were informed of the purpose of the research via telephone and chose to be interviewed via telephone. None of the respondents chose to be interviewed via video call. The researchers were available to answer any questions about the study before and after the interviews. Two members of the research team who conducted the interviews standardized the narrative interview techniques before proceeding with the data collection. The semi-structured interview questions resulted in detailed narratives, which were audio-recorded with the participants' verbal consent. The interviews lasted an average of 30 min, and notes were taken during the interviews to outline the key points of the narratives for a later analysis. The interviews continued until data saturation, which was indicated by the absence of new information, and resulted in 18 interviews from a group of 24 nurses who were eligible to be interviewed.

#### 2.1.3. Analysis

The data analysis was performed according to Colaizzi's method [26]. All interviews were transcribed verbatim, and each researcher independently examined the transcripts. Additionally, the transcripts included descriptions of what happened during the interview and paraverbal aspects (e.g., pauses and voice inflections). then, significant statements and key concepts were extracted from the narratives and discussed by the research team. As a first step, this process generated lists of central themes identified by the individual researchers. Next, all the researchers compared the interview data through their different perspectives, interpretations, and experiences to validate the research findings. Following a joint discussion, the central themes were identified. Although the interviews were conducted in Italian, after analyzing the transcripts, the nurses' quotations were translated into English.

#### 2.1.4. Ethics approval of research

The participants joined and voluntarily and anonymously participated in the study. All participants were clearly informed of the guarantee of anonymity in accordance with Italian privacy law (D.Lgs. n. 196/2003, D.Lgs. n. 101/2018), as well as their right to withdraw from the study at any time without penalty. The nurses provided verbal informed consent before the interview and after receiving an explanation of the study objectives and answers to any questions from the researchers. The interviews were recorded with the participants' consent, and each participant was assigned a unique sequential identification code to ensure anonymity (ID). This study adhered to the ethical principles of the Declaration of Helsinki and the General Data Protection Regulation (EU) 2016/679 (GDPR). Due to the non-interventional nature of the study and in the absence of any involvement of therapeutic medications, no formal approval was required. Currently, in Italy, ethical approval is not required for non-interventional studies (law 211/2003), as they are not defined as medical/clinical research. In this regard, approval by a Medical Ethics Review Committee was not required.

## 3. Results

### 3.1. Demographics

The study included a sample of 18 nurses who worked in mental health settings in southern Italy. The participants' ages ranged from 27 to 65 years, with a mean age of 49.72 years. The majority of the participants (55.56%) were male (10 nurses). The total number of years working as a nurse ranged from 3 to 35 years (mean 23.16 years). Only 22.22% (4 nurses) had worked less than 10 years, whereas 66.67% (12 nurses) had worked more than 20 years. The nurses in the sample had worked in mental health for an average of 15 years (range 1–34 years), and 66.67% (12 nurses) for at least 10 years. The average length of the interviews was 24 min, 22 seconds. The detailed results are presented in Table 2.

**Table 2.** Interviewers' characteristics.

**Participant ID**	**Gender**	**Age**	**Years working as a nurse**	**Years working as a mental health nurse**
ID1	M	65	34	34
ID2	M	50	25	11
ID3	M	31	6	6
ID4	M	57	30	30
ID5	M	63	35	28
ID6	M	49	24	1
ID7	F	62	41	10
ID8	F	51	29	20
ID9	M	48	16	10
ID10	F	51	20	2
ID11	F	35	6	1
ID12	F	59	30	15
ID13	M	51	30	30
ID14	M	56	27	18
ID15	F	58	35	32
ID16	F	27	3	1
ID17	M	27	3	1
ID18	F	55	23	20

### 3.2. Nurses' experiences of violence in mental health care

The nurses shared their aggression experiences during nursing practice in mental health settings. In this section, we describe some of the most critical incidents experienced by the nurses and referred to in their narratives.

ID8: “I will tell you about this episode of aggression after which I was off work for six months [due to injury]. The patient was very quiet; I went [to her room], I fed her, and I went back to the ward, and I told my colleagues: “You know Mrs. X ate the entire yogurt today; she was really good, no fuss”. I finished saying these words, and she [the patient] came from behind and grabbed me by my hair. She dragged me three meters. Was she angry with me? No, she was not angry with me. If she had attacked me in the room while I was feeding her, it would have been even worse (...) my colleagues took her [off me] after she dragged me by my hair, and they couldn't pull her off”.

ID9: “An ugly episode happened when I was defending a doctor who was cornered by a patient, a former boxer, by the way, who was hitting him. We have intervened with our colleague. He tore off my uniform and left me in my underwear; the colleague also lost his glasses”.

ID11: “I refused a cigarette to a drug-addicted patient, telling him in a proper way, i.e. without being aggressive, that it was not appropriate for him to smoke at that time [late at night], and unfortunately, he reacted immediately with his hands, without giving me time to explain why. He pushed me, pulled my hair, and pulled me in various ways. Luckily nothing serious happened... I was injured for twenty days”.

ID13: “It was an attack from behind, I was grabbed by the neck, I managed to wriggle out and get free... I remember a week later that it triggered a panic attack, I couldn't speak, I couldn't explain myself, and words came out slurring. That was the first episode and I was definitely... disoriented...”.

### 3.3. Main themes and sub-themes

Five main themes related to experiences of violence in a mental health context were identified during nurses' interviews. These themes include the following: (1) feelings about the violence experienced; (2) the effects of the violent incidents on nurses; (3) features of the mental health setting related to the phenomenon of the assault; (4) the care and organizational aspects to prevent the assault ; and (5) the care in psychiatric settings during the COVID-19 pandemic. All themes were further divided into three sub-themes, except for theme 4, which was divided into four sub-themes. A summary of the themes and sub-themes is presented in Table 3.

**Table 3.** Main themes and sub-themes of nurses' narratives.

**Themes**	**Subthemes**	**Interviewees**
1. Feelings about the violence experienced	1.1 Feelings of helplessness and defeat	ID1, ID3, ID6, ID11, ID13, ID15
	1.2 Emotions of anger and fear	
	1.3 Coping mechanisms	
2. The effects of violent incidents on nurses	2.1 Nurses' self-assessment of their approach to patient	ID3, ID7, ID8, ID10, ID13, ID14
	2.2 Working with high levels of attention	
	2.3 Repercussions of nurses as individuals	
3. Features of the mental health setting related to the phenomenon of the assault	3.1 Critical care issues related to pathology	ID2, ID5, ID9, ID12, ID14, ID18
	3.2 Understanding how to communicate with psychiatric patients	
	3.3 The patient's ‘tolerance’ of violence	
4. The care and organizational aspects to prevent the assault	4.1 Care strategies for preventing aggression	ID1, ID2, ID4, ID7, ID9, ID12, ID16, ID17
	4.2 Support from colleagues in the psychiatry team	
	4.3 Safety and staff shortage	
	4.4 Need for specific training	
5. The care in psychiatric settings during the COVID-19 pandemic	5.1 Nurses' perceptions of assaults	ID2, ID7, ID8, ID9, ID11, ID12
	5.2 The danger of contagion	
	5.3 Family visits and aggression	

**Theme 1. Feelings about the violence experienced**

This theme highlights the nurses' feelings about the violence they experienced, which were described as negative feelings and emotions. Additionally, adaptation strategies were used to cope with the situation experienced.

*Sub-theme 1.1. Feelings of helplessness and defeat*

In the following interviews, the nurses described the feelings aroused by the violent incidents, bringing out discomfort and regret for not having been able to prevent this from happening.

ID3 – “The emotion I felt was regret (...) it really gives me a sense of helplessness, of frustration. Maybe if I had reacted in another way, [the patient] would not have done this”.

ID13: “For me, it is a defeat when a patient or a person becomes violent. The fact is that I failed to work properly or at least failed to recognize some situations in which the patient becomes so violent”.

*Sub-theme 1.2. Emotions of anger and fear*

The emotions most reported by the participants were anger at how the interaction that led to aggression unfolded and a fear of the aggression experienced, even after the incident.

D11: “Definitely when it happened at the moment, I was afraid... then anger because I could have reacted differently. It was also my mistake in the sense that I was alone at the time, and I should not have refused a cigarette to a drug-addictive patient. So, anger because I could have dealt with it differently, and fear for what happened afterwards.”

ID15 - “I remember especially when I had small children, before I left for work, I would kiss them. I had the feeling that I would never come back. Every day, when I leaved to go to whatever shift I had to work, I kissed my husband and the children and (...) I said inside me ‘maybe I'll see you again tonight... maybe’, I was always afraid I wouldn't come back.”

*Sub-theme 1.3. Coping mechanisms*

The respondents use strategies to cope with aggression (e.g., self-control) and adapt to the difficulties they experience daily in mental health work settings. This helps them to cope better with such situations and to protect their personal environment.

ID1 - “Right from the start, I promised myself one thing: from the first day I set foot in the psychiatrist, the problems of the ward remain within the ward, when that door closes for me it's over so, I don't drag my problems home.”

ID6 - “I deal with situations as they arise and I resolve them as they arise, after that you move on. (...) The thing that really helped me a lot was self-control, to show that in front of them they have a person who knows his business and is determined (...) because unfortunately, in this type of pathology with aggression, it is counterproductive to be aggressive in response.

**Theme 2. The effects of violent incidents on nurses**

The episodes of violence led nurses to be more careful with psychiatric patients and to critically evaluate the correctness of their care approach. This is also in relation to the negative consequences for themselves because of the violence.

*Sub-theme 2.1. Nurses' self-assessments of their approach to patient*

The nurses critically reviewed what happened and attempted to identify any actions or attitudes that may have contributed to or triggered the patient's aggression. An attempt emerged to better handle violent situations.

ID13 - “sometimes you have even critical rethinking about your actions; it also gives you anger when you recognize that you were wrong or that the patient just does not listen to you and if he had listened to you, that situation could also have been avoided. Even restraint gives you anger because you failed to make [the patient] listen to you.”

ID14 - “personally I try to see where I may have gone wrong... not immediately, because maybe immediately I am a bit pissed off but then I try to see where I could have done differently...” You go, see, and say, ‘I did the right thing.’ Maybe even the way I said it...it's something beyond aggression, it's a disappointment.”

*Sub-theme 2.2. Work with high levels of attention*

The respondents are aware that their behavior can influence the occurrence of violence, but at the same time, they are aware that a psychiatric patient can be unpredictable. They believe that it is crucial to maintain high levels of attention to their behaviors and to what is happening around them while working in a psychiatry setting.

ID3 - “There has been a big increase in attention and development of senses, especially with hearing. When I'm at work, I concentrate a lot on listening; even if I'm doing something, I try to understand what's going on in different rooms, from every little noise. Then, when I came [to work] in psychiatry, the first thing they told me was that in order to work here I have to get to think where the ‘normal’ man doesn't think, for example, that a window could be opened with the handle of a door... and instead I had to think about that too.”

ID10 - “(...) we have to deal with particular patients and therefore we have to be very careful about what we say and how we take these patients because many things can be avoided. But it is also true that some things [assaults] happen independently of the nurse or the doctor; it can happen.

*Sub-theme 2.3. Repercussions of nurses as individuals*

The violence experienced has important consequences on the lives of nurses, not only as professionals, but also as individuals. The repercussions are on mood, interactions with others, and, of a physical nature, the outcomes in their daily life outside work.

ID7 - “Well... it's normal that the [working] day, I carry it with me at home as well. I definitely carry it with me the discomfort and the discontent. It's not true that you stamp [your exit from work] and leave everything here, absolutely not, at least not for me.

ID8 - “(...) last year I had an injury [due to an assault by a patient] where I practically still have problems today. I can't move my arm anymore. I'm always having difficulties. I have to do cycles of therapy. I've spent a lot of money on physiotherapy, and I'm still doing it.”

**Theme 3. Features of the mental health setting related to the phenomenon of the assault**

The interviewees are aware of the clinical care specificities of the patients they assist in the mental health setting and how necessary it is to be able to use appropriate communication techniques when interacting with them. A form of tolerance emerged to aggression related to the patient's pathology.

*Sub-theme 3.1. Critical care issues related to pathology*

The narratives show that the operational difficulties and aggressiveness experienced by the professionals are often inextricably linked to the patients' psychiatric pathology. The nurses are aware that the patient's actions and reactions are not predictable if they depend on the patient's pathology (e.g., hallucinations).

ID9 - “Patients come in for compulsory health treatment who don't want to hear reason, agitated for a thousand reasons, they put their hands on you, they just don't differentiate between doctor, nurse, social worker, and other patients. They also rage a lot psychologically; they look for every aspect of your personality; they sometimes manage to probe it to hurt you, and they succeed. But most of the time, we suffer physical violence, just beatings upon beatings.”

ID14 - “(...) it always depends on how we then place ourselves in front of the patient. If you can understand this, you can understand what can trigger the aggression and almost always you can avoid it... it is rare an aggression without reason... unless it is a person with visual hallucinations and/or also auditory so, he hears or sees things that according to him are a danger... Maybe, he sees them through us; someone [among the patients] also saw the devil when my colleague entered the room. (...) you always have to evaluate many things.”

*Sub-theme 3.2. Understanding how to communicate with a psychiatric patient*

Communicating well with the patient can calm the patient down, avoid misunderstandings, and prevent aggression. Additionally, correct communication methods are acquired through experience and become part of the care approaches to patients with mental health problems.

ID12 - “(...) talk to them in a calm tone, be polite, pretend not to hear when they swear at you or say swear words, just as if you hadn't heard them, always of course maintaining a calm tone or to try to instill even a certain calmness and not irritate them. Experience and years of working with psychiatric patients teach you a way, a behavior for treating this patient”.

ID18 - “...a lack of communication (...) leads to misunderstanding and then to conflict and the next step is violence, aggression”.

*Sub-theme 3.3. The patient's ‘tolerance’ of violence*

The patient's sick condition may lead him/her to offend healthcare personnel and engage in violent acts toward them. Nurses exhibit understanding and tolerate such situations as they recognize that they are there to assist them. In contrast, such a tolerance is not applicable to the relatives of patients who exhibit aggression toward the staff.

ID5 - “... even if the patient tells you that you are stupid, that you don't understand anything, the first thing you say is “I have to react”; but you don't, you have to think about it and try to calm the patient down, calm him down. You have to start from that, you have to remember that you are not in the street, but you are assisting people who are sick”.

ID2 - “The patient you can also understand, one is sick and maybe reacts, (...) the patient also lets go. You have to avoid the escalation of violence, so you have to try to meet them, so don't show aggression. (...) If [the aggression] happens by relatives it stresses me out a lot, it makes me nervous, it makes you a little bit harder to go to work because then you think it will happen again, then I feel bad about it, I don't know, it's also subjective, it doesn't slip away easily.

**Theme 4. The care and organizational aspects to prevent the assault**

This theme highlights factors that help counteract assaults. Nurses use care strategies daily to prevent assaults and consider team support to be crucial. The respondents considered it essential to work in a safe environment with adequate staffing levels and trained personnel to care for the psychiatric patients.

*Sub-theme 4.1. Care strategies for preventing aggression*

The sub-theme reveals care strategies that nurses have put into practice to prevent situations that may result in violence toward them. Interventions or tricks may seem simple, but can solve problems, mitigate critical situations, or avoid dangerous circumstances.

ID9 - “Sometimes the patient gets up [at night] because they want to smoke. I make them smoke, avoid a bad discussion at two or three o'clock in the morning. Another very frequent source of discussion is coffee. When we [nurses] put the coffee pot in the morning, outside, there is a line of patients knocking and saying, ‘but can I have a drop of coffee?’ Discussions ensue, which lasted throughout the day. What do we do now? We have a tin of barley coffee, we make them. They are satisfied, and you have solved the problem. They are also trivial problems, but argument ensues, violence ensues, a fight ensues, among other things, over coffee or a cigarette.”

ID12 - “(...) in certain situations [urgent hospitalization] I try to keep a safe distance, I try not to get too close, slowly... Then, as the therapy is done and they start after a week to come back [feel better] (...) It gave me a lot of experience: not to enter the room alone, especially in agitated patients in compulsory health treatment or even in other patients who are particularly agitated and have had manifestations of violence.”

*Sub-theme 4.2. Support from colleagues in the psychiatry team*

Teamwork is crucial in mental health settings to counter the occurrence of violent situations. Individual professional skills and experiences of aggression are available to all, and solutions are shared. The contributions of experienced colleagues are important to better handle situations before they become violent.

ID4 - “(...) try a little bit to refine our [the team's] resources to deal with them [violent situations] always in the best possible way and to try to prevent these things from transcending. Every experience is then placed at the service [of colleagues], and it is discussed, and solutions are found all together as a team.”

ID16 - “I also work with colleagues who have a lot of experience, so they know how to handle these people and how to calm them down at times when it may seem they are about to become violent.”

*Sub-theme 4.3. Safety and staff shortage*

Nurses often complain about the shortage of ward staff. This has a negative impact on care provision and on the level of safety that professionals perceive while working. The presence of a guard/vigilante makes them feel safer than the risk of being subjected to violence while working.

ID2 - “...maybe they make new recruitments and then other [workers] leave for other reasons, so there are always people are missing. Now, for example, the number of social health workers has increased, but we have always had very few. So, you also have to do their work. (...) But there has been a change. We have a security guard who was not there before. Now we can manage certain situations more easily. I feel even safer.

ID7 - ‘... here at work you can never feel safe, never... never let your guard down. Also, we don't have a vigilante at all. This is quite serious because we often work with some colleagues and therefore there are also logistical difficulties. Between one room and another, a corridor separates us. So, because [patients] are unpredictable, what hasn't happened in 20 years [attacks] may happen in two moments, or it will never happen.

*Sub-theme 4.4. Need for specific training*

Training is considered essential for working in mental health care settings; however, the interviewees reported that newly recruited nurses did not receive sufficient training. Therefore, it is almost always necessary to proceed with training in the ward to prepare them to adequately handle the psychiatric patient and the situations most at risk of aggression.

ID1 - “... most of the time, they [new colleagues] are poorly trained. We try to do training here [in the ward]: we say ‘remember to check if he/she is taking the therapy’, ‘never turn your back to the patients’, ‘always check that the doors are closed (certain doors of course)’, ‘if you have to open the front door because they rang, always check that the patient is not one meter away, invite him/her to move away’. Unfortunately, training at a general level does not exist, so we perform training directly on the ward.”

ID17 - “we also had no training before coming to work in this service ...”

**Theme 5. The care in psychiatric settings during the COVID-19 pandemic**

In general, aggression did not decrease during the COVID-19 pandemic, despite the respondents reporting that there were fewer patients to care for. Sometimes the patients were more aggressive (e.g., missed family visits), and sometimes they were less aggressive because they were worried about a possible contagion. Additionally, the nurses were worried about being infected during normal care and handling violent incidents (e.g., torn personal protective equipment)

*Sub-theme 5.1. Nurses' perceptions of assaults*

For the participants, the number of assaults decreased during the lockdown period (as of 9 March 2020) as admissions and interactions with patients decreased; however, in general, the incidents remained constant during the pandemic. The rules imposed to prevent a contagion (e.g., nasal swab for SARS-CoV-2) intensified the patient aggression, while concerns about a contagion probably occasionally counteracted this reaction.

ID8 - “The COVID-19 didn't really change things, there are still verbal and physical aggressions. They have also worsened because then [patients] have to stay so long in the tent waiting for the swab, they get annoyed, you have to swab them, they don't stay still...”

ID9 - “with the pandemic, the patient coming to the ward was very quiet and very worried so he didn't even think about using his hands; he thought about his condition both psychiatrically and possibly with COVID-19. [Incidents of violence] (...) decreased during the lockdown period because the ward was empty anyway, (...) but even now, that we have just over seven patients [admitted], it's the same as before, it's the same, nothing changes.

*Sub-theme 5.2. The danger of contagion*

The fear of infection during the nursing practice also emerges because of the particular conditions of some patients and the concomitant lack of medical devices. In particular, the nurses expressed concern about the possible management of episodes of physical violence that could lead to the removal or breakage of personal protective equipment.

ID7 - “(...) we were afraid at that time. We were afraid that something might have happened during the pandemic. We were here, on the front line without masks, we didn't have any medical equipment, there was nothing to protect us, we were very much out in the open with people who often live on the streets without proper hygiene, and kids with pneumonia problems of their own. So, every cough was like hearing thunder, you know? We were scared in short.”

ID12 - “I was worried because many times this type of patient is very aggressive toward us and toward other patients, and maybe it is needed to block he/she. So, as we don't have any results yet if the patient is negative for COVID-19, they grab we and, just like they tore our uniforms, they can also tear our overalls, masks, and whatever else [protective]. The difficulty of the type of patient gave me a lot to think about to also find strategies to deal with this even more particular and riskier situation.”

*Sub-theme 5.3. Family visits and aggression*

The nurses reported that not being able to receive visits from family members increased the patient aggressiveness. This directly involves family members who complain about the rules implemented to prevent a contagion and are perceived by nurses as more aggressive than before the COVID-19 pandemic.

ID11 - “... with COVID-19, the number of admissions has decreased, and so it is easier to follow them [the patients]. Before the pandemic, we used to have 15–16 patients; now we have about 6–7–8, so they are more manageable but a bit more aggressive during the pandemic because they can't have visitors. They feel alone, and let's say they have less freedom. It's a stress that you add to the pathology, and so they are more aggressive”.

ID2 - “...with patients, violence incidents are not increased. The only thing is that when the patient arrives, he/she has to stay in the tent until we have the nasal swab report for SARS-CoV-2, which can take up to four hours, so he/she has to be managed in the tent. On the other hand, with relatives, the situation is a bit worse because many do not accept that only one relative can enter [in the ward]. They always complain that they have to queue at the entrance because they perform triage. In some cases, they were even quite aggressive”.

## 4. Discussion

Violence against healthcare workers is a pervasive problem in the healthcare sector, especially in psychiatric settings. In particular, the nurses who work in these settings are at a high risk of experiencing physical and verbal abuse, which can have profound consequences on their physical and mental well-being [27]. This study explored the experiences of nurses who worked in psychiatric settings in Italy, thereby examining the violence they encountered, its impact on their well-being and nursing practice, and the coping strategies they used. In addition, the possible influence of the COVID-19 pandemic on violence towards nurses during this period and the reasons that may have led to an increase in incidents were investigated.

The study identified five key themes related to the nurses' experiences of violence in psychiatric settings.

In relation to the first theme, the nurses described a range of negative emotions in response to the violence they experienced, including discouragement, defeat, anger, and fear. These emotions were often accompanied by a sense of helplessness. The nurses reported the emotional weight of the violence, and emphasized their feelings of inadequacy and frustration in preventing such incidents. They expressed regret at their inability to effectively anticipate or manage these situations. The violence triggered intense feelings of anger, which resulted from the perceived injustice of the situation and the sense of violation [28]. Additionally, the nurses reported feeling anxious, even after the incident had passed. Such negative feelings have also been reported in other studies in the literature [29],[30], with a particular reference to the emotion of fear following the assault experienced.

To cope with these difficult experiences, nurses used various coping strategies [31], including self-control and emotional detachment. This helped them to manage the emotional impact and protect their personal well-being [32]. Similarly, in a study in Turkey [33], psychiatric nurses developed coping skills for violence in order to deal with its devastating effects. This situation increased their powers of observation and creative solutions, thus enabling them to effectively manage not only work-related crises, but also those related to their own lives.

Regarding theme 2, namely the impact of violence, the aggression went beyond the immediate incident and left a lasting impact on the nurses' personal and professional lives. The nurses reported feeling insecure and stressed, and their job satisfaction and morale were often negatively affected. In some cases, the violence resulted in physical injury and psychological trauma. Similar aspects were reported in the study by Lim et al. [29], where nurses who experienced workplace violence tended to feel more stressed than others. However, the nurses who participated in our study were committed to using coping strategies such as self-reflection [25], thereby critically analysing their actions and interactions to identify potential triggers of violence. They sought to continually improve their ability to deal with difficult situations. Furthermore, nurses are aware that their behavior can influence the occurrence of violence, but they also recognise the unpredictability of psychiatric patients. They emphasised the importance of maintaining a high level of vigilance to protect themselves and others, as reported in other studies in the literature [29],[33].

It is believed that the nurses' work experience enables them to develop unique skills and competences to prevent violent episodes (e.g., always ‘listening’ to what is happening in the different areas of the psychiatric ward, even when they were engaged in other activities). In this way, a new practical approach to preventing such situations emerges from difficult experiences. Unfortunately, however, violence has far-reaching consequences, thereby affecting the personal lives and well-being of nurses beyond their professional role, and negatively affecting their mood, relationships, and physical health [25]. As similarly reported in the study by Küçük Öztürk [33], aggression also has a very serious impact on the nurses' family life, work performance, and quality of life, particularly on their physical, mental, and social health.

Theme 3 highlights the participants' awareness of the specificity of the mental health context, where violent events are expected due to the nature of the patient's health problem. They are tolerant of the aggression they receive [31],[32], thus recognising that it often stems from the patient's underlying pathology. This is also found in the study by Soenen et al. [34], where the nurses and psychologists considered aggression from the psychiatric patient to be an integral part of the work and therefore tolerated. Furthermore, the nurses recognise the unique clinical and care needs of psychiatric patients and emphasise the importance of effective communication techniques to manage interactions. In fact, effective communication can reassure patients, therey often avoiding misunderstandings and preventing aggression [9],[35].

One of the most important contributions of this study concerns what the nurses reported doing to prevent aggression towards themselves. Indeed, the fourth theme encompasses the precautions and strategies used when caring for the psychiatric patient. The precautions to prevent violent situations include keeping a safe distance, not entering the patient's room alone, and never having the patient behind them. Additionally, the nurses reported that they used caring strategies such as giving in to the patients' easy requests (e.g., cigarettes), and not refusing the patient something but finding a compromise (e.g., giving the patient a cup of barley coffee instead of asking for the regular cup of coffee). It is believed that such specific precautions and strategies, which nurses reported as effective and useful in everyday practice, should become part of interventions specifically designed to prevent violent incidents. In this regard, a recent systematic review [36],[37] indicated that the interaction between the patient and the professional caused aggression in 40% of cases. These interactions are poor communication, a lack of empathy or respect, a lack of shared decision making in the team, restrictions for the patient, setting limits, and denying something to the patient. Additonally, it was found that, according to the nurses interviewed, the individual professional skills are strategic for the whole care team, and it is therefore important that experiences of aggression are made available to all and that solutions are shared. In this regard, Ferri et al. [38] emphasised that effective training in the management of violent incidents starts with a good collaboration and communication between staff.

Another problem that has emerged is the shortage of staff on the wards in relation to the number of patients admitted [12]. This has a negative impact on the delivery of care and on the level of safety that staff perceived in their work. In fact, according to Weltens et al. [36], the management must ensure that the maximum capacity of beds occupied on the ward (relative to the staff on duty) is not exceeded, as this is associated with an increased risk of aggressive episodes [38]. In addition, our interviews show that the presence of a guard/vigilante would make nurses feel safer with regard to the risk of experiencing violence at work [9]. Finally, education and training is seen as an essential resource for working in mental health settings; however, the respondents reported that training for newly recruited nurses was inadequate. Therefore, trainings on psychiatric wards is needed to prepare them to adequately deal with patients and situations most at risk of aggression [39].

Another interesting contribution that emerged from this study concerned the reasons that exacerbated aggression during the COVID-19 pandemic in mental health settings. Indeed, the fifth and final theme relates to the participants' perceptions of the factors that contributed to violent events during the COVID-19 pandemic. Although some reported a decrease in physical aggression, they also reported an increase in verbal aggression. Notably, the participants reported a decrease in aggression during the first lockdown period (March 2020) due to fewer admissions and interactions with patients. However, as the pandemic progressed, the aggression levels either returned to pre-pandemic levels or even worsened. This may be related to the fact that violence is often exacerbated by emergencies [40]. This trend seems to be consistent with the fact that while fear of a contagion probably counteracted aggression, it was exacerbated by the rules and procedures imposed to prevent a SARS-CoV-2 infection. Indeed, a systematic review and meta-analysis on the subject [10] suggested an increased risk of aggression towards all healthcare workers during the COVID-19 pandemic, with the rate of physical violence being more than twice as high among nurses than among doctors (13% *vs*. 5%). The nurses reported that waiting for services and activities were among the elements most likely to provoke patient aggression.

An example is the patient who first waited in a tent outside the psychiatric ward to take the nasal swab examination for SARS-CoV-2, and then to know the outcome before being admitted to psychiatry. Another reason that frequently triggered the psychiatric patient's aggression was the restrictions on their family members' access for visits. However, restrictions on family visits also contributed to the aggressiveness of the family members themselves, who were perceived by the nurses as more anxious and more aggressive than in the pre-pandemic period. This is in line with Somani et al.'s findings [12] showing that restrictions on family visits were one of the factors that contributed to increased incidents of violence during the COVID-19 pandemic. Other factors reported, such as staff shortages and lack of resources, led to increased incidents of. In this regard, the nurses interviewed in our study also highlighted some problems related to the risk of infection during the pandemic period when dealing with physically aggressive patients. In fact, the lack of personal protective equipment (PPE) during the early period of the pandemic and the risk of it being removed or damaged during violent episodes increased their concerns.

### 4.1. Limitations and future directions

This study provides valuable insights into the experiences of nurses who deal with patient aggression in psychiatric settings. However, it is critical to recognize the limitations of this study. The first limitation is inherent to the nature of qualitative studies. The small sample size limits the generalizability of the results to a broader population of nurses who work in psychiatric settings. However, the 18 nurses represent a relatively large number of participants for qualitative studies.

The second limitation was the need to comply with the legal restrictions in force in Italy during the pandemic emergency, which meant that interviews could only be conducted by telephone or video call. All the participants chose to be interviewed by telephone, so it was never possible to integrate the verbal messages with the participants' non-verbal language (e.g., facial expressions). This may have made the interaction with the interviewer more difficult and affected the quality of the data collected compared to a face-to-face or video call interview.

A third limitation was that the interviews were conducted in Italian, and the nurses' quotations were translated into English for this paper. The translation of direct quotations from one language to another can change the way someone reads and perceives excerpts. However, the text was proofread by an expert native speaker to ensure the accuracy of the translation.

Moreover, this study primarily explored the experiences of aggression and their perceived impact on the nurses' well-being. Future studies should examine the long-term effects of experiences of aggression on the well-being and professional lives of nurses. In addition, future research could consider exploring the physicians' perspectives to contribute to a complete overview of this phenomenon.

Future studies should address these limitations to inform more effective prevention interventions and support strategies for nurses in psychiatric settings.

## 5. Conclusions

This study explored nurses' experiences of workplace violence in mental health settings. The results showed that the nurses tolerated violence when it was perpetrated by the patient, despite the negative consequences for them as workers and as individuals. Although nurses have experienced negative feelings and physical injuries as a result of aggression, they still experience feelings of helplessness and defeat and consider such incidents inevitable when they were directly related to the patient's psychiatric pathology. Understanding the reasons why aggression occurs could help prevent predisposing situations. Mental health nurses should implement personal and staff strategies to prevent assault to improve patient care and protect themselves from such incidents. Targeted interventions, which are adapted to the characteristics of each psychiatric care setting, should be implemented to prevent violence, such as those used by the nurses who participated in this study (e.g., avoiding the onset of problematic situations and share the experience of the attack with colleagues to help prevent similar incidents.). This approach, together with the creation of a safer working environment by healthcare organizations where professionals work, could improve their health, work performance, and the effectiveness of psychiatric care.

## Use of AI tools declaration

The authors declare they have not used Artificial Intelligence (AI) tools in the creation of this article.
